# Bis[4-(di­methyl­amino)­pyridinium] tetra­chlorido­cuprate(II)

**DOI:** 10.1107/S1600536813028006

**Published:** 2013-10-19

**Authors:** Sofiane Bouacida, Rafika Bouchene, Amina Khadri, Ratiba Belhouas, Hocine Merazig

**Affiliations:** a Unité de Recherche de Chimie de l’Environnement et Moléculaire Structurale, CHEMS, Faculté des Sciences Exactes, Université Constantine 1 25000, Constantine, Algeria; b Département Sciences de la Matière, Faculté des Sciences Exactes et Sciences de la Nature et de la Vie, Université Oum El Bouaghi, Algeria

## Abstract

The asymmetric unit of the title salt, (C_7_H_11_N_2_)_2_[CuCl_4_], comprises half a tetrahedral tetra­chlorido­cuprate anion, being located on a twofold axis, and a protonated 4-(di­methyl­amino)­pyridine cation. The geometry around the Cu^II^ ion is highly distorted with the range of Cl—Cu—Cl angles being 94.94 (1)–141.03 (1)°. The crystal structure is stabilized by N—H⋯Cl and C—H⋯Cl hydrogen bonds. In the three-dimensional network, cations and anions pack in the lattice so as to generate chains of [CuCl_4_]^2−^ anions separated by two orientations of cation layers, which are inter­locked through π–π stacking contacts between pairs of pyridine rings, with centroid–centroid distances of 3.7874 (7) Å.

## Related literature

For general background to organic-inorganic systems, see: Bouacida (2008 ▶). For related 4-di­methyl­amino­pyridinium metal(II) chloride salts, see: Khadri *et al.* (2013 ▶). For the geometry of four-coordinated tetra­halocuprate(II) ions, see: Awwadi *et al.* (2007 ▶); Choi *et al.* (2002 ▶); Diaz *et al.* (1999 ▶); Haddad *et al.* (2006 ▶); Harlow *et al.* (1975 ▶); Marzotto *et al.* (2001 ▶); Parent *et al.* (2007 ▶).
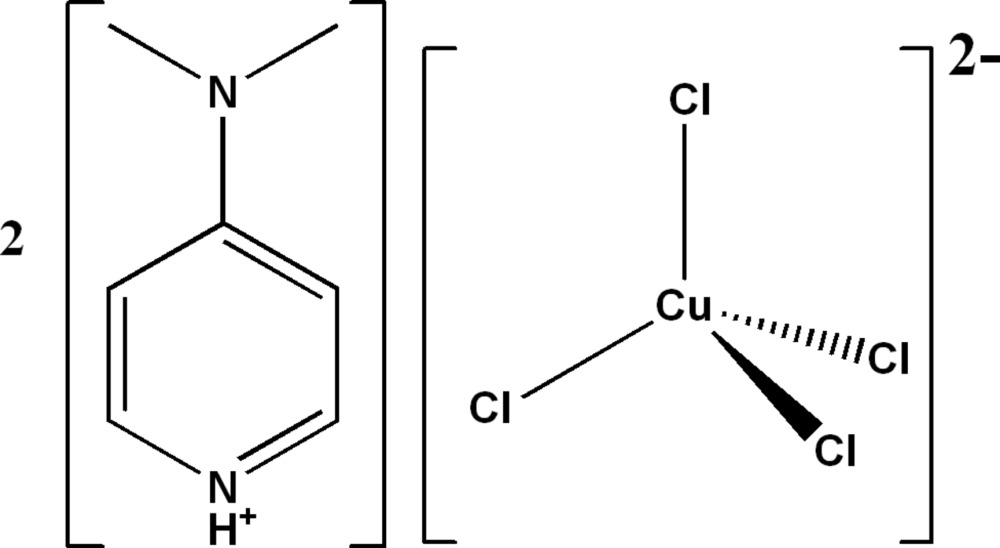


## Experimental

### 

#### Crystal data

(C_7_H_11_N_2_)_2_[CuCl_4_]
*M* *_r_* = 451.71
Monoclinic, 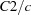

*a* = 12.3750 (8) Å
*b* = 12.1901 (8) Å
*c* = 14.1713 (9) Å
β = 115.023 (1)°
*V* = 1937.1 (2) Å^3^
*Z* = 4
Mo *K*α radiation
μ = 1.68 mm^−1^
*T* = 150 K
0.13 × 0.12 × 0.10 mm

#### Data collection

Bruker APEXII CCD diffractometer
Absorption correction: multi-scan (*SADABS*; Sheldrick, 2002 ▶) *T*_min_ = 0.675, *T*_max_ = 0.747
12787 measured reflections
3895 independent reflections
3389 reflections with *I* > 2σ(*I*)
*R*_int_ = 0.017

#### Refinement

*R*[*F* ^2^ > 2σ(*F* ^2^)] = 0.021
*wR*(*F* ^2^) = 0.059
*S* = 1.05
3895 reflections
107 parameters
H-atom parameters constrained
Δρ_max_ = 0.55 e Å^−3^
Δρ_min_ = −0.22 e Å^−3^

### 

Data collection: *APEX2* (Bruker, 2011 ▶); cell refinement: *SAINT* (Bruker, 2011 ▶); data reduction: *SAINT*; program(s) used to solve structure: *SIR2002* (Burla *et al.*, 2005 ▶); program(s) used to refine structure: *SHELXL97* (Sheldrick, 2008 ▶); molecular graphics: *ORTEP-3 for Windows* (Farrugia, 2012 ▶) and *DIAMOND* (Brandenburg & Berndt, 2001 ▶); software used to prepare material for publication: *WinGX* (Farrugia, 2012 ▶).

## Supplementary Material

Crystal structure: contains datablock(s) I. DOI: 10.1107/S1600536813028006/bq2389sup1.cif

Structure factors: contains datablock(s) I. DOI: 10.1107/S1600536813028006/bq2389Isup2.hkl

Additional supplementary materials:  crystallographic information; 3D view; checkCIF report

## Figures and Tables

**Table 1:** Selected bond lengths (Å)

Cu1—Cl1	2.2487 (3)
Cu1—Cl2	2.2588 (3)

**Table 2:** Hydrogen-bond geometry (Å, °)

*D*—H⋯*A*	*D*—H	H⋯*A*	*D*⋯*A*	*D*—H⋯*A*
N2—H2⋯Cl1	0.86	2.55	3.2264 (11)	136
N2—H2⋯Cl2	0.86	2.55	3.2760 (10)	143
C2—H2*A*⋯Cl1^i^	0.93	2.67	3.5790 (11)	167
C5—H5⋯Cl2^ii^	0.93	2.80	3.6501 (11)	152
C11—H11*B*⋯Cl2^iii^	0.96	2.82	3.6850 (13)	150

Symmetry codes: (i) 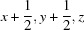; (ii) 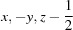; (iii) 
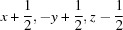
.

**Fig. 1.**
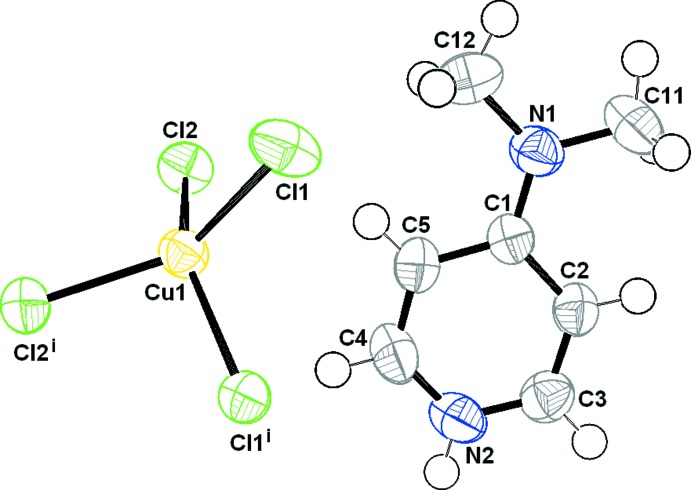
A view of molecule structure of (I) with the atom-labelling scheme. Displacement ellipsoids are drawn at the 50% probability level.

**Fig. 2.**
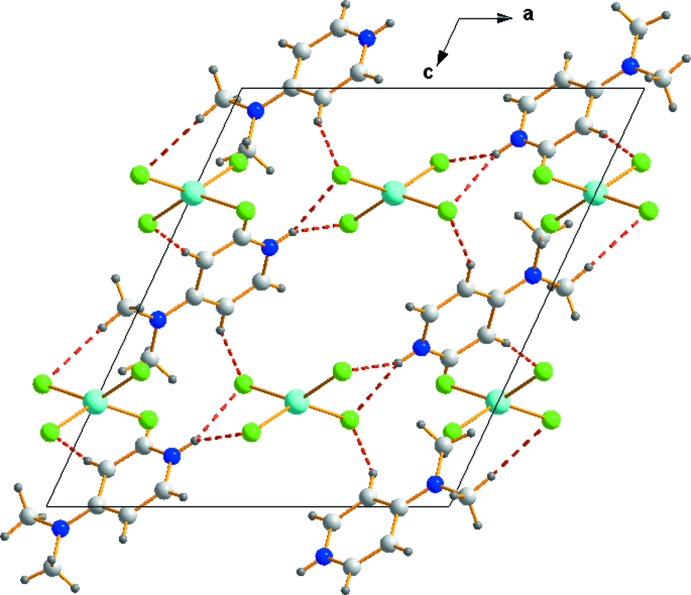
(Brandenburg & Berndt, 2001) Partial packing viewed via b axis showing structure as alternating layers of CuCl4 tetrahedral and protonated 4-Dimethylaminopyridine along the c axis and Hydrogen bonds interactions [N—H···Cl and C—H···Cl], as dashed lines.

**Fig. 3.**
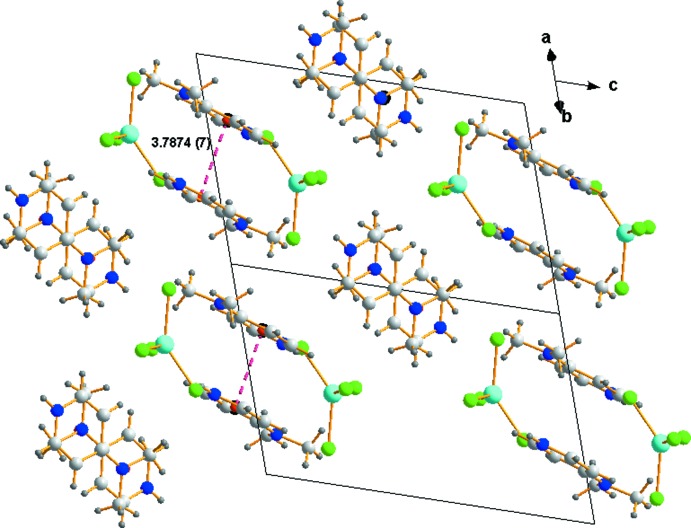
(Brandenburg & Berndt, 2001) Partial packing of (I) showing π-π stacking interactions as red dashed lines.

## supplementary crystallographic information

### 1. Comment

The role of weak intermolecular interactions in the stabilization of hybrid organic-inorganic systems is one of the main targets of our investigation in crystal engineering study (Bouacida, 2008). In continuation of our recent research on 4-dimethylaminopyridinium (HDMAP) metal halide salts (Khadri *et al.*, 2013), the X-ray crystal structures of new one with tetrachlorocuprate (II) anion is reported.

Electronic subshell d9 of Cu(II) is responsible for distortions of symmetry of the coordination polyhedron. This deals with the Jahn-Teller effect. The shape of the four-coordinated tetrahalocuprate (II) ions changes from square planar (Harlow *et al.*, 1975) to distorted tetrahedral (Diaz *et al.*, 1999) and the geometry of [Cu*X*_4_]^2-^ species is influenced by the crystal-packing forces resulted from the size and the form of counter cations (Diaz *et al.*, 1999; Parent *et al.*, 2007), hydrogen bonding to cations (Haddad *et al.*, 2006; Marzotto *et al.*, 2001; Choi *et al.*, 2002), and halide-halide interactions in solid (Awwadi *et al.*, 2007). The degree of distortion of [Cu*X*_4_]^2-^ coordination polyhedra is determined by the mean value of the flattering or *trans*-angle θ. The asymmetric unit of the title compound, shown in figure 1, contains one half of the copper chloride salt, the other half is generated by a twofold rotation axis (4 e) on which Cu(II) is situated. The [CuCl_4_]^2-^ ions are highly distorted with a mean *trans* angle of 141.02° as a result of hydrogen bonding interactions with two nearly planar HDMAP cations (0.0295 Å mean deviation). The pyridinium nitrogen forms bifurcated hydrogen bond to two chloride ligands Cl1 and Cl2 and the created organic-inorganic hybrid compound (Fig. 2) is further assembled by C—H···Cl hydrogen bonding interactions (Table 2). In the three dimension network (Fig. 3), cations and anions pack in the lattice to generate chains of [Cu*X*_4_]^2-^ anions separated by two orientations of cation layers which are interlocked through π-π stacking contacts between pairs of pyridine rings with distances centroid-centroid of 3.7874 (7) Å. All these interactions bonds link the layers together, forming a three-dimensional network and reinforcing the cohesion of ionic structure. Additionel hydrogen bond parameters are listed in table 1.

### 2. Experimental

4-dimethylaminopyridine and CuCl_2_·2H_2_O in a molar ratio of 1:1 were dissolved in sufficient acidified water (HCl, 37%). Evaporation of obtained solution at room temperature yields yellow crystals of the title compound after one week which crystals suitable for X-ray diffraction were carefully isolated.

### 3. Refinement

All H atoms were localized on Fourier maps but introduced in calculated positions and treated as riding on their parent atoms (C and N) with C—H = 0.96 Å (methyl) or C—H = 0.93 Å (aromatic) N—H = 0.86 Å and with *U*_iso_(H) = 1.2 *U*_eq_(C_aryl_ or N )and *U*_iso_(H) = 1.5 *U*_eq_(C_methyl_).

### Crystal data

**Table:**

(C_7_H_11_N_2_)_2_[CuCl_4_]	*F*(000) = 924
*M**_r_* = 451.71	*D*_x_ = 1.549 Mg m^−^^3^
Monoclinic, *C*2/*c*	Mo *K*α radiation, λ = 0.71073 Å
Hall symbol: -C 2yc	Cell parameters from 6282 reflections
*a* = 12.3750 (8) Å	θ = 2.5–34.7°
*b* = 12.1901 (8) Å	µ = 1.68 mm^−^^1^
*c* = 14.1713 (9) Å	*T* = 150 K
β = 115.023 (1)°	Cube, yellow
*V* = 1937.1 (2) Å^3^	0.13 × 0.12 × 0.10 mm
*Z* = 4	

### Data collection

**Table:**

Bruker APEXII CCD diffractometer	3895 independent reflections
Radiation source: sealed tube	3389 reflections with *I* > 2σ(*I*)
Graphite monochromator	*R*_int_ = 0.017
φ and ω scans	θ_max_ = 34.7°, θ_min_ = 2.5°
Absorption correction: multi-scan (*SADABS*; Sheldrick, 2002)	*h* = −19→19
*T*_min_ = 0.675, *T*_max_ = 0.747	*k* = −18→18
12787 measured reflections	*l* = −22→22

### Refinement

**Table:**

Refinement on *F*^2^	Primary atom site location: structure-invariant direct methods
Least-squares matrix: full	Secondary atom site location: difference Fourier map
*R*[*F*^2^ > 2σ(*F*^2^)] = 0.021	Hydrogen site location: inferred from neighbouring sites
*wR*(*F*^2^) = 0.059	H-atom parameters constrained
*S* = 1.05	*w* = 1/[σ^2^(*F*_o_^2^) + (0.0283*P*)^2^ + 0.9756*P*] where *P* = (*F*_o_^2^ + 2*F*_c_^2^)/3
3895 reflections	(Δ/σ)_max_ = 0.001
107 parameters	Δρ_max_ = 0.55 e Å^−^^3^
0 restraints	Δρ_min_ = −0.22 e Å^−^^3^

### Special details

**Table:**

Geometry. Bond distances, angles *etc*. have been calculated using the rounded fractional coordinates. All su's are estimated from the variances of the (full) variance-covariance matrix. The cell e.s.d.'s are taken into account in the estimation of distances, angles and torsion angles
Refinement. Refinement of *F*^2^ against ALL reflections. The weighted *R*-factor *wR* and goodness of fit *S* are based on *F*^2^, conventional *R*-factors *R* are based on *F*, with *F* set to zero for negative *F*^2^. The threshold expression of *F*^2^ > σ(*F*^2^) is used only for calculating *R*-factors(gt) *etc*. and is not relevant to the choice of reflections for refinement. *R*-factors based on *F*^2^ are statistically about twice as large as those based on *F*, and *R*- factors based on ALL data will be even larger.

### Fractional atomic coordinates and isotropic or equivalent isotropic displacement parameters (Å^2^)

**Table:**

	*x*	*y*	*z*	*U*_iso_*/*U*_eq_	
N1	0.43961 (8)	0.14134 (8)	−0.05308 (7)	0.0225 (2)	
N2	0.24689 (8)	0.09752 (8)	0.12016 (7)	0.0250 (3)	
C1	0.37610 (8)	0.12756 (8)	0.00275 (7)	0.0179 (2)	
C2	0.39370 (8)	0.19580 (8)	0.08966 (7)	0.0202 (2)	
C3	0.32794 (9)	0.17919 (9)	0.14518 (8)	0.0229 (3)	
C4	0.22731 (9)	0.03122 (9)	0.03815 (9)	0.0259 (3)	
C5	0.28831 (9)	0.04393 (8)	−0.02169 (8)	0.0222 (2)	
C11	0.53277 (10)	0.22510 (10)	−0.02403 (9)	0.0286 (3)	
C12	0.41514 (11)	0.07546 (10)	−0.14600 (9)	0.0280 (3)	
Cu1	0.00000	0.05740 (1)	0.25000	0.0173 (1)	
Cl1	0.07732 (2)	−0.06512 (2)	0.17693 (2)	0.0251 (1)	
Cl2	0.15211 (2)	0.17785 (2)	0.29261 (2)	0.0211 (1)	
H2	0.20730	0.08760	0.15680	0.0300*	
H2A	0.45010	0.25170	0.10860	0.0240*	
H3	0.33920	0.22490	0.20110	0.0270*	
H4	0.17090	−0.02440	0.02220	0.0310*	
H5	0.27260	−0.00200	−0.07840	0.0270*	
H11A	0.49760	0.29640	−0.02970	0.0430*	
H11B	0.57180	0.22070	−0.06980	0.0430*	
H11C	0.59000	0.21310	0.04640	0.0430*	
H12A	0.42520	−0.00080	−0.12750	0.0420*	
H12B	0.46940	0.09570	−0.17540	0.0420*	
H12C	0.33470	0.08820	−0.19620	0.0420*	

### Atomic displacement parameters (Å^2^)

**Table:**

	*U*^11^	*U*^22^	*U*^33^	*U*^12^	*U*^13^	*U*^23^
N1	0.0217 (4)	0.0262 (4)	0.0203 (4)	−0.0016 (3)	0.0097 (3)	0.0010 (3)
N2	0.0215 (4)	0.0318 (5)	0.0241 (4)	−0.0019 (3)	0.0120 (3)	0.0016 (3)
C1	0.0164 (4)	0.0182 (4)	0.0172 (4)	0.0003 (3)	0.0052 (3)	0.0015 (3)
C2	0.0200 (4)	0.0194 (4)	0.0192 (4)	−0.0025 (3)	0.0063 (3)	−0.0007 (3)
C3	0.0226 (4)	0.0250 (5)	0.0200 (4)	0.0010 (3)	0.0079 (3)	−0.0012 (3)
C4	0.0217 (4)	0.0265 (5)	0.0276 (5)	−0.0067 (4)	0.0087 (4)	−0.0001 (4)
C5	0.0210 (4)	0.0215 (4)	0.0217 (4)	−0.0036 (3)	0.0067 (3)	−0.0032 (3)
C11	0.0245 (5)	0.0341 (6)	0.0282 (5)	−0.0058 (4)	0.0122 (4)	0.0049 (4)
C12	0.0320 (5)	0.0322 (6)	0.0216 (4)	0.0056 (4)	0.0132 (4)	0.0010 (4)
Cu1	0.0164 (1)	0.0169 (1)	0.0192 (1)	0.0000	0.0082 (1)	0.0000
Cl1	0.0269 (1)	0.0203 (1)	0.0353 (1)	−0.0061 (1)	0.0200 (1)	−0.0087 (1)
Cl2	0.0205 (1)	0.0197 (1)	0.0232 (1)	−0.0035 (1)	0.0095 (1)	−0.0029 (1)

### Geometric parameters (Å, º)

**Table:**

Cu1—Cl1^i^	2.2487 (3)	C2—C3	1.3653 (16)
Cu1—Cl2^i^	2.2588 (3)	C4—C5	1.3618 (17)
Cu1—Cl1	2.2487 (3)	C2—H2A	0.9300
Cu1—Cl2	2.2588 (3)	C3—H3	0.9300
N1—C1	1.3409 (15)	C4—H4	0.9300
N1—C12	1.4606 (15)	C5—H5	0.9300
N1—C11	1.4627 (16)	C11—H11A	0.9600
N2—C3	1.3498 (15)	C11—H11B	0.9600
N2—C4	1.3507 (15)	C11—H11C	0.9600
N2—H2	0.8600	C12—H12B	0.9600
C1—C2	1.4246 (13)	C12—H12C	0.9600
C1—C5	1.4217 (15)	C12—H12A	0.9600
			
Cl1^i^—Cu1—Cl2^i^	94.94 (1)	C3—C2—H2A	120.00
Cl1—Cu1—Cl2^i^	141.03 (1)	C2—C3—H3	119.00
Cl1—Cu1—Cl2	94.94 (1)	N2—C3—H3	120.00
Cl1—Cu1—Cl1^i^	96.76 (1)	N2—C4—H4	119.00
Cl1^i^—Cu1—Cl2	141.03 (1)	C5—C4—H4	119.00
Cl2—Cu1—Cl2^i^	98.91 (1)	C1—C5—H5	120.00
C1—N1—C12	120.89 (10)	C4—C5—H5	120.00
C1—N1—C11	120.68 (9)	H11A—C11—H11B	109.00
C11—N1—C12	118.41 (10)	H11A—C11—H11C	110.00
C3—N2—C4	120.68 (10)	N1—C11—H11A	109.00
C4—N2—H2	120.00	N1—C11—H11B	109.00
C3—N2—H2	120.00	N1—C11—H11C	109.00
C2—C1—C5	116.81 (9)	H11B—C11—H11C	109.00
N1—C1—C2	121.57 (9)	H12B—C12—H12C	109.00
N1—C1—C5	121.62 (9)	N1—C12—H12A	109.00
C1—C2—C3	120.08 (9)	N1—C12—H12B	109.00
N2—C3—C2	121.05 (10)	N1—C12—H12C	109.00
N2—C4—C5	121.52 (11)	H12A—C12—H12B	109.00
C1—C5—C4	119.84 (9)	H12A—C12—H12C	109.00
C1—C2—H2A	120.00		
			
C11—N1—C1—C2	2.21 (15)	N1—C1—C2—C3	−179.66 (10)
C11—N1—C1—C5	−177.56 (10)	C5—C1—C2—C3	0.11 (14)
C12—N1—C1—C2	−175.95 (10)	N1—C1—C5—C4	178.72 (10)
C12—N1—C1—C5	4.28 (16)	C2—C1—C5—C4	−1.06 (15)
C4—N2—C3—C2	−1.18 (16)	C1—C2—C3—N2	1.00 (16)
C3—N2—C4—C5	0.20 (17)	N2—C4—C5—C1	0.93 (17)

Symmetry code: (i) −*x*, *y*, −*z*+1/2.

### Hydrogen-bond geometry (Å, º)

**Table:**

*D*—H···*A*	*D*—H	H···*A*	*D*···*A*	*D*—H···*A*
N2—H2···Cl1	0.86	2.55	3.2264 (11)	136
N2—H2···Cl2	0.86	2.55	3.2760 (10)	143
C2—H2*A*···Cl1^ii^	0.93	2.67	3.5790 (11)	167
C5—H5···Cl2^iii^	0.93	2.80	3.6501 (11)	152
C11—H11*B*···Cl2^iv^	0.96	2.82	3.6850 (13)	150

Symmetry codes: (ii) *x*+1/2, *y*+1/2, *z*; (iii) *x*, −*y*, *z*−1/2; (iv) *x*+1/2, −*y*+1/2, *z*−1/2.

## References

[bb1] Awwadi, F. F., Willett, R. D. & Twamly, B. (2007). *Cryst. Growth Des.* **7**, 624–632.

[bb2] Bouacida, S. (2008). PhD thesis, Montouri–Constantine University, Algeria.

[bb3] Brandenburg, K. & Berndt, M. (2001). *DIAMOND* Crystal Impact, Bonn, Germany.

[bb4] Bruker (2011). *APEX2* and *SAINT* Bruker AXS Inc., Madison, Wisconsin, USA.

[bb5] Burla, M. C., Caliandro, R., Camalli, M., Carrozzini, B., Cascarano, G. L., De Caro, L., Giacovazzo, C., Polidori, G. & Spagna, R. (2005). *J. Appl. Cryst.* **38**, 381–388.

[bb6] Choi, S.-N., Lee, Y.-M., Lee, H.-W., Kang, S. K. & Kim, Y.-I. (2002). *Acta Cryst.* E**58**, m583–m585.

[bb7] Diaz, I., Fernandes, V., Belsky, V. K. & Martinez, J. L. (1999). *Z. Naturforsch. Teil B*, **54**, 718–724.

[bb8] Farrugia, L. J. (2012). *J. Appl. Cryst.* **45**, 849–854.

[bb9] Haddad, S. F., Aidamen, M. A. & Willett, R. D. (2006). *Inorg. Chim. Acta*, **359**, 424–432.

[bb10] Harlow, R. L., Wells, W. J., Watt, G. W. & Simonsen, S. H. (1975). *Inorg. Chem.* **14**, 1786–1772.

[bb11] Khadri, A., Bouchene, R., Bouacida, S., Merazig, H. & Roisnel, T. (2013). *Acta Cryst.* E**69**, m190. 10.1107/S160053681300603X PMC3629474 23633992

[bb12] Marzotto, A., Clemente, D. A., Benetollo, F. & Valle, G. (2001). *Polyhedron*, **20**, 171–177.

[bb13] Parent, A. R., Landee, C. P. & Turnbull, M. M. (2007). *Inorg. Chim. Acta*, **360**, 1943–1953.

[bb14] Sheldrick, G. M. (2002). *SADABS* University of Göttingen, Germany.

[bb15] Sheldrick, G. M. (2008). *Acta Cryst.* A**64**, 112–122. 10.1107/S0108767307043930 18156677

